# Cytotoxic Activities and the Allantoinase Inhibitory Effect of the Leaf Extract of the Carnivorous Pitcher Plant *Nepenthes miranda*

**DOI:** 10.3390/plants11172265

**Published:** 2022-08-31

**Authors:** En-Shyh Lin, Cheng-Yang Huang

**Affiliations:** 1 Department of Beauty Science, National Taichung University of Science and Technology, Taichung City 403, Taiwan; 2 Department of Biomedical Sciences, Chung Shan Medical University, Taichung City 402, Taiwan; 3 Department of Medical Research, Chung Shan Medical University Hospital, Taichung City 402, Taiwan

**Keywords:** *Nepenthes miranda*, anticancer, allantoinase, PC-9 pulmonary adenocarcinoma, B16F10 melanoma, 4T1 mammary carcinoma, antioxidation, antibacterial, dihydroorotase

## Abstract

*Nepenthes* are carnivorous pitcher plants that have several ethnobotanical uses, such as curing stomachache and fever. Here, we prepared different extracts from the stem, leaf, and pitcher of *Nepenthes miranda* to further investigate their pharmacological potential. The leaf extract of *N. miranda* obtained by 100% acetone (*N. miranda*-leaf-acetone) was used in this study to analyze the cytotoxic activities, antioxidation capacity, antibacterial activity, and allantoinase (ALLase) inhibitory effect of this plant. The cytotoxic effects of *N. miranda*-leaf-acetone on the survival, apoptosis, and migration of the cancer cell lines PC-9 pulmonary adenocarcinoma, B16F10 melanoma, and 4T1 mammary carcinoma cells were demonstrated. Based on collective data, the cytotoxic activities of *N. miranda*-leaf-acetone followed the order: B16F10 > 4T1 > PC-9 cells. In addition, the cytotoxic activities of *N. miranda*-leaf-acetone were synergistically enhanced when co-acting with the clinical anticancer drug 5-fluorouracil. *N. miranda*-leaf-acetone could also inhibit the activity of ALLase, a key enzyme in the catabolism pathway for purine degradation. Through gas chromatography–mass spectrometry, the 16 most abundant ingredients in *N. miranda*-leaf-acetone were identified. The top six compounds in *N. miranda*-leaf-acetone, namely, plumbagin, lupenone, palmitic acid, stigmast-5-en-3-ol, neophytadiene, and citraconic anhydride, were docked to ALLase, and their docking scores were compared. The docking results suggested plumbagin and stigmast-5-en-3-ol as potential inhibitors of ALLase. Overall, these results may indicate the pharmacological potential of *N. miranda* for further medical applications.

## 1. Introduction

Phytochemicals obtained from plant extracts play a very prominent role as traditional medicines with many ethnopharmacological uses [1,2]. Some active ingredients from plant extracts have been introduced as promising anticancer drugs, such as vincristine, vinblastine, and paclitaxel [3,4]. Some plant extracts can be used in combination with clinical anticancer drugs to increase the efficacy of chemotherapy. One significant advantage of using natural extracts against cancer cells is their multitargeted modes of action [4,5,6]. The different active ingredients in a plant extract can provide significant polypharmacological and synergistic effects for cancer therapies [6].

*Nepenthes* are carnivorous pitcher plants with passive pitcher-shaped traps, a unique morphological and anatomical feature linked to carnivory [7]. The genus *Nepenthes* includes almost 120 species. To adapt to poor soils, *Nepenthes* attract, catch, retain, and digest prey such as insects to obtain supplemental nutrients such as nitrogen and phosphorus. For medical use, *Nepenthes* exhibit several ethnobotanical properties, such as curing stomachache and fever [7]. Furthermore, some *Nepenthes* extracts have significant anticancer and antibacterial activities [7,8,9,10]. Thus, it is worth determining targets inhibited by *Nepenthes* extracts, such as the extract of *Nepenthes miranda* used in this study, a new cultivar of a humanmade hybrid of *N. maxima* and *N. northiana* that exhibits unique physiological properties [11], for further medical applications.

Allantoinase (ALLase; EC 3.5.2.5) plays an essential role in the catabolism pathway for purine degradation [12]. ALLase is present in a wide variety of organisms and catalyzes the reversible conversion of allantoin to allantoic acid by hydrolytic cleavage of the five-member hydantoin ring. This ALLase-catalyzed reaction is a key process in the biosynthesis of ureide, which is required for the utilization of nitrogen in purine-derived compounds [13]. Structurally, ALLase possesses a binuclear metal center in which two Fe ions are bridged by a post-translationally carbamylated lysine [14,15]. From a biochemical point of view [16,17], ALLase [18] is a member of the cyclic amidohydrolase family [19,20], which also includes dihydroorotase (DHOase) [21,22,23,24,25,26,27], dihydropyrimidinase (DHPase) [28,29,30,31,32,33,34,35], hydantoinase [36,37,38], and imidase [39,40,41]. Some of these amidohydrolases are suggested as chemotherapeutic targets for anticancer, antimicrobial, and antimalarial drug developments because of their involvement in the key reactions of nucleotide biosynthesis. Thus, exploiting the new inhibitors against these targets is of considerable interest for drug development.

Antimicrobial drug resistance is an increasing threat to global public health [42]. Growing concern worldwide in human and animal infections caused by antibiotic-resistant microorganisms has spurred the interest of the scientific community in antibiotic development. Multidrug resistance among ESKAPE organisms [42,43,44], i.e., *Enterococcus faecium*, *Staphylococcus aureus*, *Klebsiella pneumoniae*, *Acinetobacter baumannii*, *Pseudomonas aeruginosa*, and *Enterobacter* spp., is of particular concern because they are responsible for many life-threatening hospital infections [42]. Antibiotic-resistant *K. pneumoniae* are a major cause of hospital- and community-acquired infections, including sepsis, liver abscesses, and pneumonia [45,46,47]. It has been established that the utilization of allantoin as a nitrogen source is a very important virulence determinant in *K. pneumoniae* for liver abscesses [48]. Thus, the pharmacological inhibition of the allantoin-degradation pathway may be useful in decreasing the virulence of *K. pneumonia*, and possibly other pathogens [48,49]. In this study, we first found the plant extract of *N. miranda* capable of inhibiting the activity of ALLase, which was a key enzyme for allantoin degradation.

The top cause of death by cancer worldwide is lung cancer [50,51,52]. Non-small cell lung cancer (NSCLC) is the most common type of lung cancer and accounts for about 85% of all lung cancers. Treatments for NSCLC include surgery, chemotherapy, radiation therapy, and targeted therapy [52]. However, NSCLCs are relatively insensitive to chemotherapy. In addition, several adverse effects caused by modern chemotherapy hinder cancer treatment and lead to other unavoidable critical disorders. Therefore, natural compounds as potential anticancer agents and alternative medicines are also being used for cancer treatment [5,53,54]. Accordingly, we examined here the cytotoxicity of *N. miranda* on the survival, migration, and apoptosis of human NSCLC PC-9 adenocarcinoma cells. In addition, the anti-PC-9 effects of the co-use of the extract of *N. miranda* in combination with other anticancer agents were also demonstrated. For comparison, the cytotoxic effects of the *N. miranda* extract against B16F10 melanoma and 4T1 mammary carcinoma cells were also demonstrated.

The chemical composition of the leaf extract of *N. miranda* was analyzed via gas chromatography–mass spectrometry (GC–MS). Through GC–MS, the 16 most abundant ingredients in the leaf extract of *N. miranda* were identified. The top four contents, plumbagin (28.52%), lupenone (11.45%), palmitic acid (5.49%), and stigmast-5-en-3-ol (5.06%), especially plumbagin and lupenone, are anticancer compounds. This might be why *N. miranda*-leaf-acetone possesses strong anticancer activities. Further studies should directly focus on determining whether and how the extract of *N. miranda* can be used as an alternative medicine.

## 2. Results

### 2.1. Antioxidant Activity

**Figure 1:**
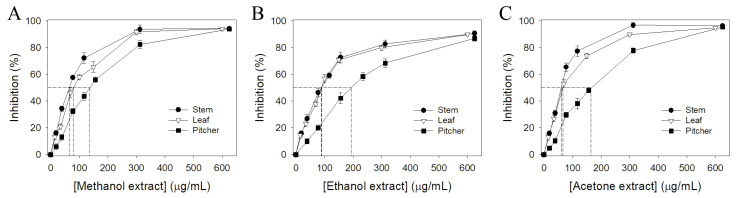
Antioxidant activity of different extracts of *N. miranda*. The antioxidant activities of extracts from stems, leaves, and pitchers prepared using (**A**) methanol, (**B**) ethanol, and (**C**) acetone were evaluated by DPPH radical scavenging assay.

Various parts of *N. miranda*, i.e., the leaf, stem, and pitcher, were collected, dried, cut into small pieces, pulverized into powder, and extracted using different solvents (water, methanol, ethanol, and acetone). Pharmacological potentials and antioxidant activity are usually correlated [55,56]. Therefore, we analyzed the antioxidant activities of different extracts of *N. miranda* using 1,1-diphenyl-2-picrylhydrazyl (DPPH) radical scavenging assay. DPPH assay is the most common method to assess the antioxidant capacity of plants. The antioxidant capacities of different *N. miranda* extracts were described by IC_50_ values. IC_50_ values of different extracts of *N. miranda* were calculated from the DPPH titration curves by determining the extract concentration needed to achieve the midpoint value for inhibition (Figure 1); a lower IC_50_ value indicated higher radical scavenging activity (Table 1). IC_50_ values of water extracts of *N. miranda* were too low to determine. As compared to their IC_50_ values, the antioxidant capacity of extracts of *N. miranda* mainly followed the order: stem > leaf > pitcher. *N. miranda*-stem-acetone showed the highest antioxidant capacity with an IC_50_ value of 59.7 ± 3.2 μg/mL. The second highest was *N. miranda*-leaf-acetone, with an IC_50_ value of 66.0 ± 2.8 μg/mL.

### 2.2. Antibacterial Activity

*K. pneumoniae*, as a major cause of hospital- and community-acquired infections, including sepsis, liver abscesses, and pneumonia [47], are dangerous ESKAPE organisms highly correlated with many life-threatening infections. We used the agar well diffusion method to assess the anti-*K. pneumoniae* activity of the different extracts of *N. miranda*. While the water extract showed no effect, the other extracts of *N. miranda* exhibited different activities for suppressing the growth of *K. pneumonia*, with the zone of inhibition ranging from 9 to 27 mm (Table 2). The anti-*K. pneumoniae* activity of extracts of *N. miranda* mainly followed the order: stem > leaf > pitcher. Acetone was the best solvent for extracting useful ingredients in inhibiting the growth of *K. pneumoniae*.

### 2.3. Cytotoxic Activity against Human PC-9 Pulmonary Adenocarcinoma

**Figure 2:**
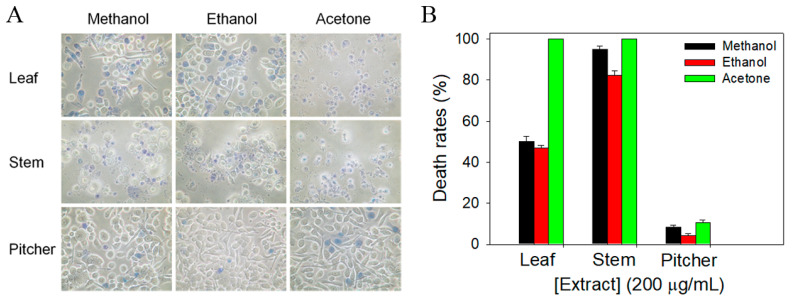
The cytotoxic effects of different *N. miranda* extracts against PC-9 cells. (**A**) Trypan blue dye exclusion staining. The cytotoxic effects of different *N. miranda* extracts against PC-9 cells were estimated with trypan blue assay after 24 h of incubation. The PC-9 cells incubated with *N. miranda*-stem-acetone and *N. miranda*-leaf-acetone of 200 μg/mL were almost dead. (**B**) The death rates of PC-9 cells. The anti-PC-9 activity of *N. miranda* extracts followed the order: acetone > methanol > ethanol.

The most frequent cause of death by cancer worldwide is lung cancer [50]. In addition, the 5-year survival rate is still very poor for patients with advanced stage. Whether extracts of *N. miranda* could cause the death of human pulmonary adenocarcinoma cells remained uninvestigated; thus, we used NSCLC PC-9 cells to evaluate the anti-cancer activities of different extracts of *N. miranda* (Figure 2). The monolayers prepared in 96-well microtitration plates for PC-9 cells were inoculated with different *N. miranda* extracts at a concentration of 200 μg/mL per well. The death rate of PC-9 cells by *N. miranda* extracts was estimated with trypan blue staining assay after 0 and 24 h of incubation (Figure 2A). The cytotoxic capacity of *N. miranda* extracts followed the order: acetone > methanol > ethanol. The water extracts of *N. miranda* did not have any cytotoxic effect on the survival of PC-9 cells (data not shown). Two acetone extracts, *N. miranda*-stem-acetone and *N. miranda*-leaf-acetone, showed significant PC-9 cell death (100%). Considering that leaves are far more abundant than stems for *N. miranda*, *N. miranda*-leaf-acetone was chosen to examine the phytochemical composition and other cytotoxic properties (see below).

### 2.4. Gas Chromatography–Mass Spectrometry (GC–MS) Analysis of N. miranda-Leaf-Acetone

**Figure 3:**
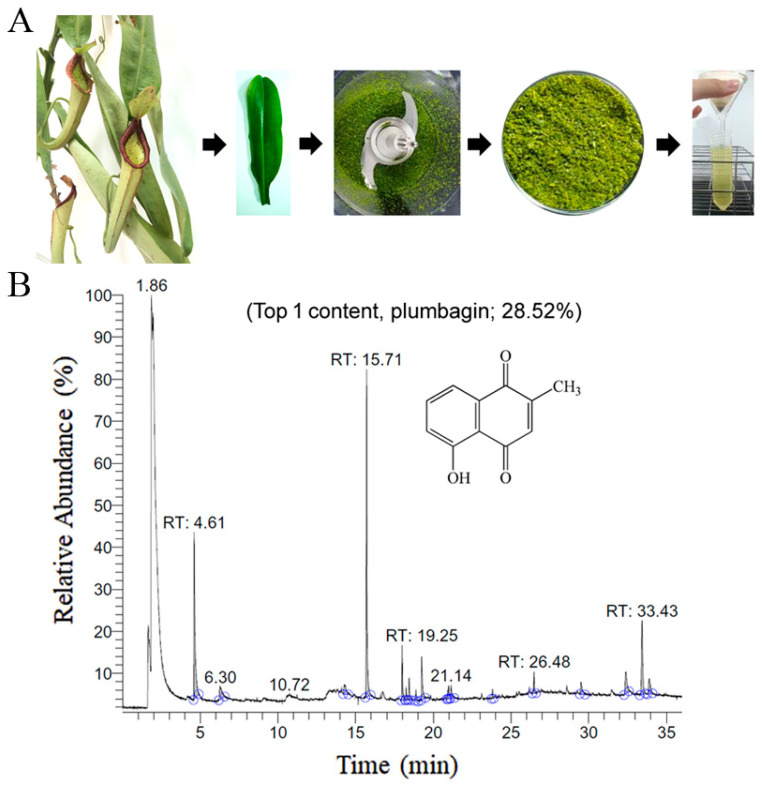
GC–MS analysis of *N. miranda*-leaf-acetone. (**A**) Preparation of the leaves extract. (**B**) GC chromatogram of compounds presents in *N. miranda*-leaf-acetone. Compounds were identified by matching generated spectra with NIST 2011 and Wiley 10th edition mass spectral libraries. Plumbagin (28.52%) was the major compound present in *N. miranda*-leaf-acetone.

GC–MS was used to detect individual compounds abundant in *N. miranda*. Given that *N. miranda*-leaf-acetone had high antioxidation activity (Table 1), anti-*K. pneumonia* (Table 2), and anti-PC 9 cells (Figure 2), we focused on determining the medicinally active ingredients in *N. miranda*-leaf-acetone (Figure 3A) through GC–MS. The GC chromatogram showed that at least 16 compounds in *N. miranda*-leaf-acetone were detected (Figure 3B). These compounds were identified by matching generated spectra with NIST 2011 and Wiley 10th edition mass spectral libraries (Table 3). The top 16 contents (>0.6%) were as follows: plumbagin (28.52%), lupenone (11.45%), palmitic acid (5.49%), stigmast-5-en-3-ol (5.06%), neophytadiene (4.72%), citraconic anhydride (3.96%), lupeol (3.52%), phytol (2.98%), melezitose (2.67%), vitamin E (2.40%), stearic acid (1.91%), linolenic acid (1.79%), squalene (1.60%), geranyl isovalerate (1.12%), (2,2-dimethyl-1,3-dioxolan-4-yl)-methyl palmitate (0.76%), and Z-7-hexadecenal (0.67%). Some of these compounds are known to possess anticancer capacities, such as plumbagin [57,58,59,60].

### 2.5. Dose-Dependent Cytotoxic Effects of N. miranda-Leaf-Acetone on the Survival and Migration of PC-9, 4T1, and B16F10 Cells

**Figure 4:**
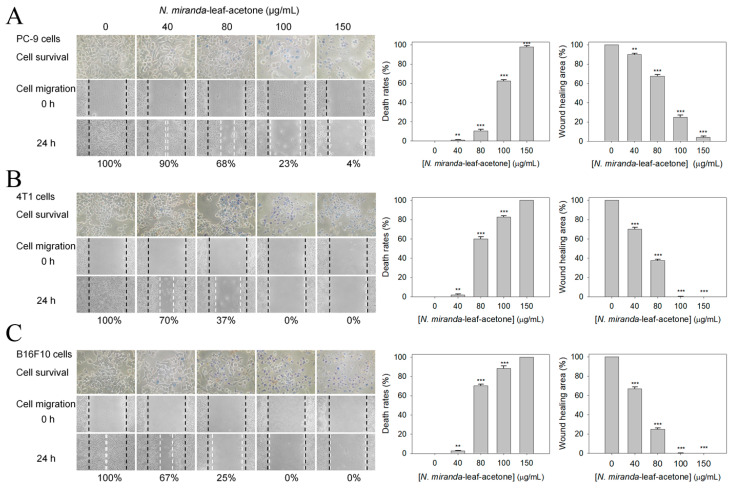
Cytotoxic effects of *N. miranda*-leaf-acetone on the survival and migration of (**A**) PC-9, (**B**) 4T1, and (**C**) B16F10 Cells. Different concentrations of *N. miranda*-leaf-acetone (0, 40, 80, 100, and 150 μg/mL) were used to demonstrate the cytotoxic effects on the survival and migration of these cancer cells. Trypan blue assay was performed to estimate the cell death rates and wound-healing assay was used to estimate the cell migration capacities. ** *p* < 0.01 and *** *p* < 0.001 compared with the control group.

PC-9 cells incubated with *N. miranda*-leaf-acetone of 200 μg/mL were almost completely killed (Figure 2). Different concentrations of *N. miranda*-leaf-acetone (0, 40, 80, 100, and 150 μg/mL) were used to demonstrate further the cytotoxic effects on the survival and migration of PC-9 cells (Figure 4A). The cytotoxic effects of *N. miranda*-leaf-acetone against 4T1 mammary carcinoma (Figure 4B) and B16F10 melanoma (Figure 4C) cells were also investigated and compared. Incubation with *N. miranda*-leaf-acetone of 0, 40, 80, 100, and 150 μg/mL caused the deaths of PC-9 cells at the rate of 0, 1, 11, 62, and 98%, respectively. The same concentrations of *N. miranda*-leaf-acetone used against 4T1 and B16F10 cells were much more efficient than against PC-9 cells. For example, incubation with *N. miranda*-leaf-acetone of 80 μg/mL caused the deaths of 4T1 and B16F10 cells at the rates of 60 and 71%, while only 11% of PC-9 cells were affected. Accordingly, *N. miranda*-leaf-acetone was also useful against 4T1 and B16F10 cells, and the cytotoxic activities of *N. miranda*-leaf-acetone followed the order: B16F10 > 4T1 > PC-9 cells.

The in vitro migration capacities of PC-9, 4T1, and B16F10 cells suppressed by *N. miranda*-leaf-acetone were estimated with a wound-healing assay (Figure 4). After different treatments, cells were incubated for 24 h to allow migration. The wound-healing areas for PC-9 cells treated with *N. miranda*-leaf-acetone of 0, 40, 80, 100, and 150 μg/mL were 100, 90, 68, 25, and 4%, respectively. *N. miranda*-leaf-acetone of 100 μg/mL could completely inhibit the migration of 4T1 and B16F10 cells. The inhibitory effects of *N. miranda*-leaf-acetone on cancer cell migration were in the order: B16F10 > 4T1 > PC-9 cells.

### 2.6. Co-Treatment of N. miranda-Leaf-Acetone with Epothilone B against PC-9 Cells

**Figure 5:**
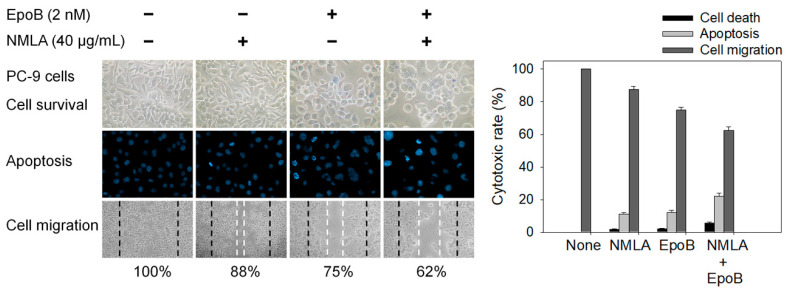
The co-cytotoxic effects of epothilone B with *N. miranda*-leaf-acetone. *N. miranda*-leaf-acetone (NMLA; 40 μg/mL) and epothilone B (EpoB; 2 nM) were used to investigate the cytotoxic effects on cell survival, migration, and apoptosis. The collective data suggested no potential synergistic cytotoxic effects.

Epothilone B [61] is a stabilizing tubulin antagonist with broad anti-tumor activity, used for the treatment of ovarian cancer, lung cancer, brain cancer, breast cancer, and gastric cancer. The principal mechanism of epothilone B is the inhibition of microtubule function [61,62]. Microtubules are essential to cell division, and epothilone B, therefore, stops cells from properly dividing. Accordingly, we investigated whether this paclitaxel-like natural product could co-act with *N. miranda*-leaf-acetone against PC-9 cancer cells. *N. miranda*-leaf-acetone of 40 μg/mL, capable of inducing a minor cytotoxic effect, was selected for this co-treatment experiment (Figure 5). The use of *N. miranda*-leaf-acetone and epothilone B (2 nM) led to 2% and 3% cell mortality, respectively. The co-treatment of *N. miranda*-leaf-acetone with epothilone B led to 5% cell mortality. This result suggested no potential synergistic cytotoxic effects because cell mortality was not obviously raised. The result from the wound-healing assay corroborated this finding. Through the Hoechst staining assay, the use of *N. miranda*-leaf-acetone, epothilone B, and co-treatment of *N. miranda*-leaf-acetone with epothilone B induced apoptosis with DNA fragmentation in PC-9 cells at the rate of 11%, 12%, and 22%, respectively. Thus, epothilone B could suppress PC-9 cells (Figure 5), but it could not produce an additional cytotoxic effect in co-treatment with *N. miranda*-leaf-acetone.

### 2.7. Co-Treatment of N. miranda-Leaf-Acetone with 5-FU Synergistically Induced Apoptosis of PC-9, B16F10, and 4T1 Cells

**Figure 6:**
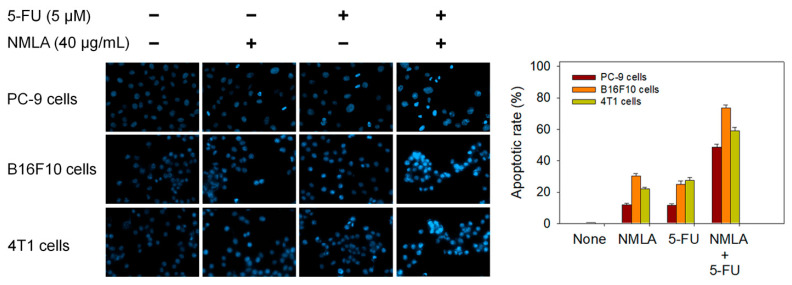
The co-cytotoxic effect of the clinical anticancer drug 5-FU with *N. miranda*-leaf-acetone. *N. miranda*-leaf-acetone (NMLA; 40 μg/mL) and 5-FU (5 μM) were used to investigate the cytotoxic effect on PC-9, B16F10, and 4T1 cells apoptosis. The collective data suggested potential synergistic anticancer effect when co-treatment.

Epothilone B and *N. miranda*-leaf-acetone individually induced PC-9 cell apoptosis (Figure 5); however, additional efficacy was not found with co-use. We then investigated whether the clinical anticancer drug 5-FU could synergistically enhance the cytotoxic activity of *N. miranda*-leaf-acetone against PC-9, B16F10, and 4T1 cells (Figure 6). Through the Hoechst staining assay, we found that the use of *N. miranda*-leaf-acetone (40 μg/mL), 5-FU (5 μM), and co-treatment of *N. miranda*-leaf-acetone with 5-FU induced apoptosis of PC-9 cells at the rate of 12%, 12%, and 49%, respectively. This result indicated a potential synergistic cytotoxic effect because the co-treatment of *N. miranda*-leaf-acetone with 5-FU could produce more DNA fragmentations (a nearly 2-fold increase) in PC-9 cells. This was also true in B16F10 and 4T1 cells. Use of *N. miranda*-leaf-acetone, 5-FU, and the co-treatment induced apoptosis at the rates of 30%, 25%, and 74% in B16F10 cells, and 22%, 27%, and 59% in 4T1 cells, respectively. The synergistic cytotoxic effect was in the order: B16F10 > 4T1 > PC-9 cells. Accordingly, we might conclude that *N. miranda*-leaf-acetone can be co-used with 5-FU for better anticancer applications. However, this speculation must be further demonstrated experimentally and clinically.

### 2.8. ALLase Inhibitory Potential

**Figure 7:**
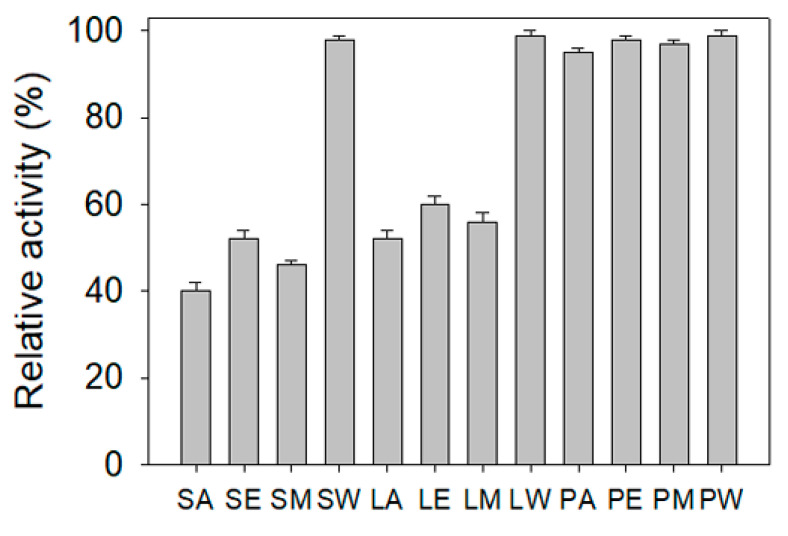
ALLase inhibitory potential. Different extracts of *N. miranda* (30 μg/mL) were used for inhibiting the activity of ALLase. S, stem; L, leaf; P, pitcher; A, acetone; E, ethanol; M, methanol; W, water.

ALLase plays an essential role in the catabolism pathway for purine degradation [12]. The ALLase-catalyzed reaction is a key process in the biosynthesis of ureide, which is required for the utilization of nitrogen in purine-derived compounds. Thus, ALLase may be a cytotoxic target. Currently, there is still no report regarding the inhibition effect of any plant extract on the activity of ALLase. In this study, we also attempted to find whether extracts from *N. miranda* could inhibit ALLase. The recombinant ALLase from *Salmonella enterica* serovar Typhimurium LT2 was hetero-overexpressed in *Escherichia coli*, purified by Ni^2+^-affinity chromatography, and used for this investigation. By using the standard assay, the inhibitory effect of different extracts from *N. miranda* on ALLase was found to be in the order: *N. miranda*-stem-acetone > *N. miranda*-stem-methanol > *N. miranda*-stem-ethanol = *N. miranda*-leaf-acetone > *N. miranda*-leaf-methanol > *N. miranda*-leaf-ethanol (Figure 7). Other *N. miranda* extracts exhibited slight or negligible inhibition effects. *N. miranda*-stem-acetone and *N. miranda*-leaf-acetone showed ALLase inhibition at the rate of 60% and 48% at a 30 μg/mL concentration, respectively. These results indicated that one or more compounds in the stem and leaf of *N. miranda* could be potential inhibitors of ALLase.

### 2.9. Molecular Docking

**Figure 8:**
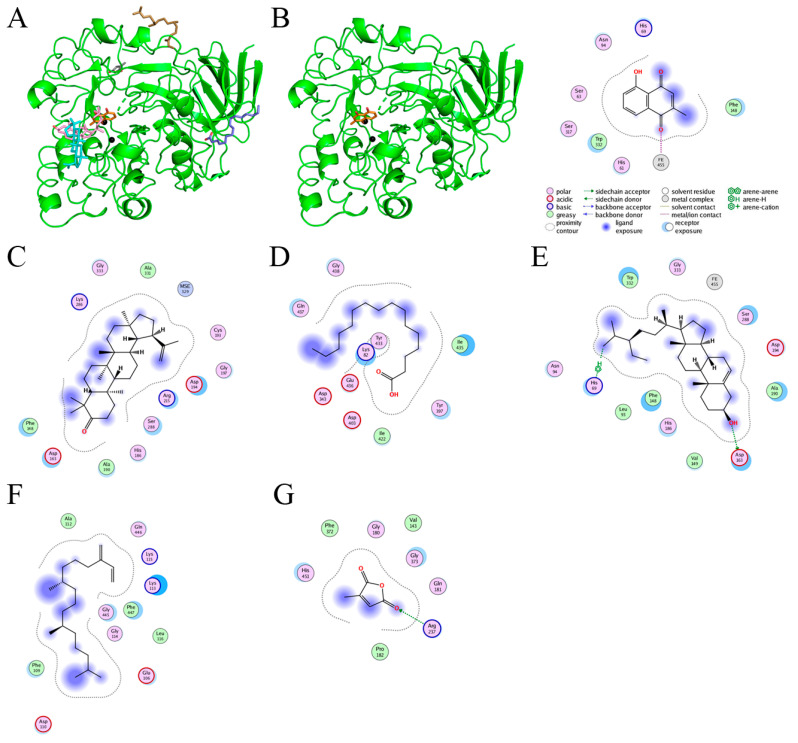
Molecular docking. (**A**) Docking results. Through MOE-Dock, ALLase–ligand binding affinities with all possible binding geometries could be predicted on the basis of the S score. The PDB ID of the used structure of ALLase (green) is 3E74. This enzyme contains a binuclear metal center. Two Fe ions in the active site are presented as black spheres. The top 6 compounds in *N. miranda*-leaf-acetone, namely plumbagin (orange), lupenone (cyan), palmitic acid (slate), stigmast-5-en-3-ol (lightpink), neophytadiene (sand), and citraconic anhydride (gray), were docked to ALLase. Only plumbagin could be docked into the active site of ALLase. (**B**) The binding mode of plumbagin to ALLase. (**C**) The binding mode of lupenone to ALLase. (**D**) The binding mode of palmitic acid to ALLase. (**E**) The binding mode of stigmast-5-en-3-ol to ALLase. (**F**) The binding mode of neophytadiene to ALLase. (**G**) The binding mode of citraconic anhydride to ALLase.

The current work identified that some *N. miranda* extracts possess anti-ALLase activity (Figure 7). It was tentatively proposed that certain compound in *N. miranda* is responsible for inhibiting the activity of ALLase and, therefore, their binding modes should be elucidated (Figure 8A). Based on our GC–MS results for *N. miranda*-leaf-acetone, their binding capacities were analyzed via the MOE (molecular operating environment)-Dock tool [63]. Through MOE-Dock, receptor–ligand binding affinities with all possible binding geometries could be predicted on the basis of the docking score (the S score). The top six compounds in *N. miranda*-leaf-acetone (Table 3), plumbagin (Figure 8B), lupenone (Figure 8C), palmitic acid (Figure 8D), stigmast-5-en-3-ol (Figure 8E), neophytadiene (Figure 8F), and citraconic anhydride (Figure 8G) were docked to ALLase (PDB ID 3E74), and their S scores were compared (Table 4). Based on the S scores, the binding capacity of these compounds was in the order: stigmast-5-en-3-ol > palmitic acid > lupenone > plumbagin > neophytadiene > citraconic anhydride. Accordingly, stigmast-5-en-3-ol, possessing the highest S score, might exhibit the greatest binding affinity to ALLase among these selected compounds.

## 3. Discussion

Cancer is one of the leading causes of human mortality [64,65]. However, cancer cells can hijack and remodel existing metabolic pathways for their survival and proliferation [66,67], making them hard to treat. Conventional cancer treatments commonly involve radiotherapy and chemotherapy but with several adverse effects, and they commonly lead to other critical disorders. Therefore, natural compounds as potential anticancer agents are also used and studied in many cancer models, both in vitro and in vivo [5,54,68]. Currently, promising plant-based anticancer medicines such as vincristine, vinblastine, and paclitaxel have been developed and used in clinical applications [3,4]. More plant-based anticancer compounds should be identified as therapeutics for pharmaceutical applications.

*Nepenthes* exhibit several ethnobotanical uses, such as curing stomachache and fever, and have fewer side effects in human use [7]. In this study, *N. miranda*-leaf-acetone was found to possess cytotoxic activities against pulmonary adenocarcinoma (PC-9 cells), skin melanoma (B16F10 cells), and mammary carcinoma (4T1 cells). The cytotoxic effect of *N. miranda*-leaf-acetone was in the order: B16F10 > 4T1 > PC-9 cells (Figure 4). In addition, the use of *N. miranda*-leaf-acetone could be combined with the clinical anticancer drug 5-FU for the synergistic cytotoxic effect, in the order: B16F10 > 4T1 > PC-9 cells. These collective data suggested that *N. miranda*-leaf-acetone could be a potential natural alternative or complementary therapy for these cancers, especially melanoma and mammary carcinoma. The active ingredients in *N. miranda*-leaf-acetone (Table 3) should be isolated and identified for further pharmacological applications.

The leaf extract of *N. miranda* may be a better alternative medicine than the stem extract, as the leaves of *N. miranda* are available in higher quantities than the stems. In addition, removing the stem for extractions kills the whole *N. miranda* plant, whereas only taking some leaves from *N. miranda* allows it to survive. Thus, *N. miranda*-leaf-acetone was suggested for further pharmaceutical use.

Multidrug-resistant pathogenic bacteria are spreading rapidly worldwide and can become untreatable [42]. *K. pneumonia* are a concerning ESKAPE organism [42,43,44]. Antibiotic-resistant *K. pneumoniae* can cause sepsis, liver abscesses, and pneumonia and are responsible for many life-threatening hospital infections [45,46,47]. The utilization of allantoin as a nitrogen source is recognized as very important to the virulence of *K. pneumoniae* [48], and, therefore, ALLase might be a promising target. We found that extracts of *N. miranda* exhibit anti-*K. pneumoniae* (Table 2) and anti-ALLase activities (Figure 7). Whether some plant-derived products from the stem and/or leaf extract of *N. miranda* may act as active antibacterial agents for human health care is worth further determining.

Many phenolic compounds occurring naturally in plants can be effective for humans in treating various disorders due to their antioxidant, anti-inflammatory, antibacterial, and anticancer activities. We found the cytotoxicity of *N. miranda*-leaf-acetone against PC-9, B16F10, and 4T1 cancer cells. Through GC–MS, the contents abundant in *N. miranda*-leaf-acetone were detected and identified (Table 3). We found that the top four contents, plumbagin (28.52%) [57,58,59,60], lupenone (11.45%) [69,70,71], palmitic acid (5.49%) [72,73,74], and stigmast-5-en-3-ol (5.06%) [75], especially plumbagin and lupenone, are anticancer compounds. This might be why *N. miranda*-leaf-acetone possesses strong cytotoxic activities. Whether these different active ingredients in *N. miranda*-leaf-acetone can provide significant polypharmacological and synergistic effects and in what ratio for cancer therapies should be elucidated.

ALLase [14,16,18,76], DHOase [21,22,23,24,25,26,27], and DHPase [28,29,30,31,32,33,34,35] are members of the cyclic amidohydrolase family [16]. Although these cyclic amidohydrolases use a similar active site and mechanism for catalysis, no substrate overlapping was observed among them [18]. However, DHOase [8] and DHPase [9] can have a common inhibitor, namely plumbagin. Recently, we solved the complex crystal structure of DHOase with plumbagin (PDB ID 7CA1) [8], and the structure revealed that plumbagin occupies the active site and prevents the substrate from entering. Plumbagin was the most abundant substance in *N. miranda*-leaf-acetone (Table 3), and a docking study indicated plumbagin capable of occupying the active site of ALLase (Figure 8 and Table 4). Accordingly, we speculated that the inhibition of ALLase by *N. miranda*-leaf-acetone mainly comes from plumbagin. We will further analyze whether plumbagin could be an inhibitor and the inhibition mode against ALLase in future research.

We also found that the stem and leaf extracts of *N. miranda* can strongly inhibit the DNA-binding activity of single-stranded DNA-binding proteins (SSB) (unpublished results). Like ALLase, SSB is also an attractive target for potential antipathogen chemotherapy because it is absolutely required for DNA replication and cell survival [77,78,79,80,81]. Recently, we have identified that the natural products myricetin [77,81] and taxifolin [78] can inhibit the activity of SSB. It is worth demonstrating which active component(s) in *N. miranda*-leaf-acetone and *N. miranda*-stem-acetone can inhibit SSB for further antibiotic developments and applications.

Previously, we solved crystal structures of DHPase [28,30], SSB [80], and DHOase [24] in a complex with 5-FU. Therefore, 5-FU may be involved in the activity regulation of these proteins. In this study, we further found that the cytotoxic effects of *N. miranda*-leaf-acetone can be enhanced synergistically when it is co-used with 5-FU (Figure 6). On the other hand, co-cytotoxic effects of *N. miranda*-leaf-acetone and epothilone B (Figure 5), a stabilizing tubulin antagonist with broad anti-tumor activity used in ovarian cancer, lung cancer, brain cancer, breast cancer, and gastric cancer, were not significant. Moreover, 5-FU is an FDA-approved anticancer drug that is widely used in clinical applications [82,83,84,85]. As a potent antimetabolite, 5-FU can cause RNA miscoding [86], inhibit DNA synthesis [86], and increase the intracellular reactive oxygen species (ROS)-related radical anion O_2_ level [84,87]. ROS can induce apoptotic cell death via a p53-dependent pathway [88,89,90]. Similarly, plumbagin can also exert anticancer activity by generating intracellular ROS and inducing apoptosis [10,60]. Thus, plumbagin, the most abundant substance in *N. miranda*-leaf-acetone, may strongly enhance the chemosensitivity of 5-FU by promoting ROS production for anticancer activity. Furthermore, 5-FU and plumbagin may also co-act to enhance cytotoxicity against cancer cells by targeting DHPase [9,28,30] and DHOase [8,24] to suppress DNA metabolism. How *N. miranda*-leaf-acetone can co-act with 5-FU and how the chemosensitivity level can be enhanced should be further elucidated.

In conclusion, we evaluated the antioxidant, anti-*K. pneumoniae*, and anti-ALLase activities of different parts (stem, leaf, and pitcher) of *N. miranda* extracts that were obtained by using methanol, ethanol, acetone, and distilled water. The cytotoxic effects of *N. miranda*-leaf-acetone on the survival, apoptosis, and migration of PC-9, 4T1, and B16F10 cancer cells were examined. The ingredients abundant in *N. miranda*-leaf-acetone were determined by GC–MS for further polypharmacological and synergistic applications. These collective results might indicate the pharmacological potentials of *N. miranda* for further clinical anticancer chemotherapies.

## 4. Materials and Methods

### 4.1. Chemicals, Cell Lines, and Bacterial Strains

All chemicals were purchased from Sigma-Aldrich (St. Louis, MO, USA) and were of analytical grade. The *E. coli* strain BL21(DE3) pLysS (Novagen, UK) was used for protein expression and purification. *K. pneumoniae* MGH 78578 [91,92,93,94] was used for the antibacterial assay. The cell lines PC-9 pulmonary adenocarcinoma, 4T1 carcinoma, and B16F10 murine melanoma were obtained from the Food Industry Research and Development Institute, Hsinchu, Taiwan [95].

### 4.2. Plant Materials and Extract Preparations

Leaves, stems, and pitchers of *N. miranda* were collected, dried, cut into small pieces, and pulverized into powder. Extractions were carried out by placing 1 g of plant powder into a 250 mL conical flask. The flask was added with 100 mL of solvents (methanol, ethanol, acetone, or distilled water) and shaken on an orbital shaker for 5 h. The resultant extract was filtered using a 0.45-μm filter and stored at −80 °C until use.

### 4.3. Determination of Antioxidant Activity by DPPH Radical Scavenging Assay

The antioxidant potential of the plant extracts was determined using a DPPH assay [96]. DPPH free radical scavenging activity was determined using the formula: %Radical scavenging activity = (Control OD − Sample OD)/Control OD × 100. The absorbance was measured at 517 nm.

### 4.4. GC-MS Analysis

Phytochemical components of *N. miranda*-leaf-acetone were determined by GC-MS [95]. The filtered sample was analyzed using Thermo Scientific TRACE 1300 Gas Chromatograph with a Thermo Scientific ISQ Single Quadrupole Mass Spectrometer system. The column used was Rxi-5ms (30 m × 0.25 mm i.d. × 0.25 μm film). The compounds discharged from the column were detected by a quadrupole mass detector. The ions were generated by the electron ionization method. The relative mass fraction of each chemical component was determined by the peak area normalization method. Compounds were identified by matching generated spectra with NIST 2011 and Wiley 10th edition mass spectral libraries.

### 4.5. Cell Culture

B16F10 and 4T1 cells were maintained as a monolayer culture in Dulbecco’s modified Eagle medium supplemented with 10% fetal bovine serum (FBS) and 4 mM L-glutamine. PC-9 cells were maintained in RPMI 1640 with 10% FBS and 2 mM L-glutamine. Cells were incubated at 37 °C in a 95% air and 5% CO_2_ incubator.

### 4.6. Trypan Blue Cytotoxicity Assay

The trypan blue cytotoxicity assay was performed to assess cell death [97]. The cancer cells (1 × 10^4^) were incubated with different extracts in a 100 μL volume. After 24 h, the cytotoxic potentiality exhibited by the extract was estimated by performing a trypan blue cytotoxicity assay. A non-cancerous HEK293 cell line was used as control cells in this cytotoxicity experiment. Incubation with *N. miranda*-leaf-acetone of 80 μg/mL caused the deaths of 4T1 and B16F10 cells at the rates of 60 and 71%, while only 3% of HEK293 cells were affected.

### 4.7. Chromatin Condensation Assay

The apoptosis in cancer cells was assayed with Hoechst 33342 staining [98]. The cells were seeded in 96-well plates at a density of 5 × 10^3^ cells per well in a volume of 200 μL of culture medium. Cells were allowed to adhere for 16 h. After different treatments, cells were incubated for an additional 24 h, washed with PBS, and stained with Hoechst dye (1 μg/mL) in the dark at RT for 10 min. Cells were imaged using the ImageXpress Pico (Molecular Devices, CA, USA). Image acquisition was performed on each well using a 20× magnification, a 6 × 6 square image scan, on the DAPI filter cubes. Image analyses were performed on the images obtained from the ImageXpress Pico instrument (Molecular Devices, CA, USA) using the CellReporterXpress Version 2 software. The apoptotic index was calculated as follows: apoptotic index = apoptotic cell number/(apoptotic cell number + nonapoptotic cell number).

### 4.8. Wound-Healing Assay

An in vitro migration (wound healing) assay was performed as described previously [99]. Briefly, the cancer cells were seeded in 24-well plates, incubated in serum-reduced medium for 6 h, wounded in a line across the well with a 200 μL pipette tip, and washed twice with the serum-reduced medium. After different treatments, cells were incubated for 24 h to allow migration.

### 4.9. Antibacterial Activities

The agar well diffusion assay was performed as described previously [100]. Colonies of *K. pneumoniae* were diluted to prepare a 0.1 McFarland standard suspension. Then, the bacteria were inoculated into sterile Petri dishes of 60 mL of Muller–Hinton agar plates. The plates were shaken gently to allow even mixing of bacterial cells and agar. All samples were dissolved in 30% DMSO to furnish 22 mg/mL. Exactly 90 μL of each extracted sample (6.0 mm diameter disc) was transferred onto the plate and incubated at 37 °C for 12 h. The diameters of the inhibition zones were calculated. Clear inhibition zones formed around the discs indicating the presence of antibacterial activity.

### 4.10. Protein Purification

The recombinant ALLase from *S. enterica* was purified as described previously [14]. Briefly, *E. coli* BL21(DE3) cells were transformed with the expression vector, and the overexpression of the expression plasmid was induced by incubating with 1 mM isopropyl thiogalactopyranoside. The protein was purified from the soluble supernatant by using Ni^2+^-affinity chromatography (HiTrap HP; GE Healthcare Bio-Sciences), eluted with buffer A (20 mM Tris–HCl, 250 mM imidazole, and 0.5 M NaCl, pH 7.9), and dialyzed against a dialysis buffer (20 mM Tris–HCl and 0.1 M NaCl, pH 7.9). The protein purity remained at >97%, as determined using SDS–PAGE (Mini-PROTEAN Tetra System; Bio-Rad, CA, USA).

### 4.11. Enzyme Assay

A rapid spectrophotometric assay was used to determine the enzymatic activity of ALLase [18]. Hydrolysis of the substrate allantoin was measured at 25 °C as the decrease in absorbance at 258 nm [76]. To start the reaction, the purified ALLase was preincubated with 1 mM MnCl_2_ for 4 min, and the protein solution was then added to a 2-mL solution containing 10 mM allantoin and 100 mM Tris–HCl at pH 8.0. Allantoin absorbs at 258 nm with an extinction coefficient of 0.0261 mM^−1^ cm^−1^. The hydrolysis of the substrate was monitored with a UV/vis spectrophotometer (Hitachi U 3300; Hitachi High-Technologies, Tokyo, Japan).

### 4.12. Molecular Docking

Through MOE-Dock [63], plumbagin, lupenone, palmitic acid, stig-mast-5-en-3-ol, neophytadiene, and citraconic anhydride were docked to ALLase (PDB ID 3E74) for their binding capacity. Their S scores and binding modes were compared. Top-ranked confirmations were analyzed.

## Figures and Tables

**Table 1:** Antioxidant activities of *N. miranda* extracts.

Solvent	IC_50_ (μg/mL)		
	Stem	Leaf	Pitcher
Methanol	66.1 ± 2.6	79.3 ± 4.0	137.2 ± 3.9
Ethanol	88.4 ± 3.8	90.2 ± 3.7	193.2 ± 5.2
Acetone	59.7 ± 3.2	66.0 ± 2.8	166.6 ± 4.5

IC_50_ values were calculated from the titration curves of the DPPH assay by determining the concentration of the extract needed to achieve the midpoint value for inhibition. Due to <50% inhibition at concentration of 600 μg/mL, we did not determine the IC_50_ values of the water extracts of *N. miranda*.

**Table 2:** Inhibition zone of *N. miranda* extracts.

Solvent	Zone of Inhibition (mm)		
	Stem	Leaf	Pitcher
Water	0	0	0
Methanol	22	21	11
Ethanol	21	16	9
Acetone	27	24	16

The water extracts of *N. miranda* did not inhibit the growth of *K. pneumoniae*.

**Table 3:** GC-MS analysis of *N. miranda*-leaf-acetone.

Peak No.	RT (min)	Compound	MF	CS	MW	Area (%)
1	15.71	Plumbagin	C_11_H_8_O_3_	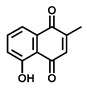	188	28.52
2	33.43	Lupenone	C_30_H_48_O	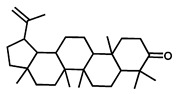	424	11.45
3	19.25	Palmitic acid	C_16_H_32_O_2_	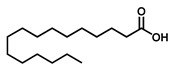	256	5.49
4	32.38	Stigmast-5-en-3-ol	C_29_H_50_O	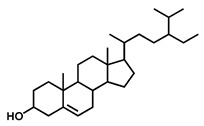	414	5.06
5	18.00	Neophytadiene	C_20_H_38_	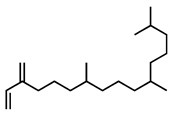	278	4.72
6	6.29	Citraconic anhydride	C_5_H_4_O_3_	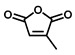	112	3.96
7	33.90	Lupeol	C_30_H_50_O	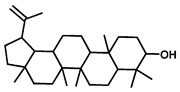	426	3.52
8	18.44	Phytol	C_20_H_40_O	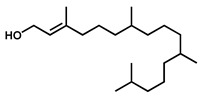	296	2.98
9	14.28	Melezitose	C_18_H_32_O_16_	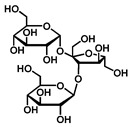	504	2.67
10	29.51	Vitamin E	C_29_H_50_O_2_	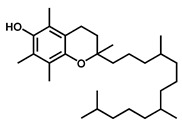	430	2.40
11	21.14	Stearic acid	C_18_H_36_O_2_	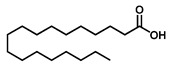	284	1.91
12	20.99	Linolenic acid	C_18_H_30_O_2_	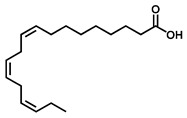	278	1.79
13	26.48	Squalene	C_30_H_50_	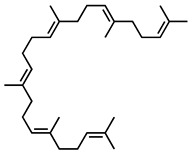	410	1.60
14	18.88	Geranyl isovalerate	C_15_H_26_O_2_	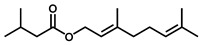	238	1.12
15	23.81	(2,2-Dimethyl-1,3-dioxolan-4-yl)-methyl palmitate	C_22_H_42_O_4_	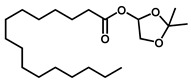	370	0.76
16	20.93	Z-7-Hexadecenal	C_16_H_30_O	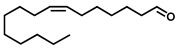	238	0.67

RT, retention time; MF, molecular formula; CS, chemical structure; MW, molecular weight.

**Table 4:** Results of the docking studies against ALLase.

Compound	S Score	Residue	Interaction	Distance (Å)	E (kcal/mol)
Plumbagin	−5.097	Fe-α	Metal	2.20	−2.2
Lupenone	−5.365	No important residue			
Palmitic acid	−5.443	No important residue			
Stigmast-5-en-3-ol	−5.689	Asp163	H-donor	2.74	−2.9
		His69	H-pi	3.78	−0.6
Neophytadiene	−5.006	No important residue			
Citraconic anhydride	−3.659	Arg237	H-acceptor	3.15	−1.0

## Data Availability

Not applicable.
